# The gut fungal and bacterial microbiota in pediatric patients with inflammatory bowel disease introduced to treatment with anti-tumor necrosis factor-α

**DOI:** 10.1038/s41598-022-10548-7

**Published:** 2022-04-22

**Authors:** Rebecka Ventin-Holmberg, Miikka Höyhtyä, Schahzad Saqib, Katri Korpela, Anne Nikkonen, Anne Salonen, Willem M. de Vos, Kaija-Leena Kolho

**Affiliations:** 1 Translational Immunology Research Program, University of Helsinki, Helsinki, Finland; 2 Faculty of Medicine and Health Technology, Tampere University, Tampere, Finland; 3 Faculty of Medicine, Human Microbiome Research Program, University of Helsinki, Helsinki, Finland; 4 University of Helsinki and Children’s Hospital, Helsinki University Hospital, University of Helsinki, Helsinki, Finland Stenbäckinkatu 11 (Skidicum),; 5 Laboratory of Microbiology, Wageningen University, Wageningen, The Netherlands

**Keywords:** Biomarkers, Microbiome

## Abstract

Pediatric inflammatory bowel disease (PIBD) is a globally increasing chronic inflammatory disease associated with an imbalanced intestinal microbiota and treated with several treatment options, including anti-tumor necrosis factor alpha (TNF-α), such as infliximab (IFX). Up to half of the patients do not respond to the drug and there are no methods for response prediction. Our aim was to predict IFX response from the gut microbiota composition since this is largely unexplored in PIBD. The gut microbiota of 30 PIBD patients receiving IFX was studied by MiSeq sequencing targeting 16S and ITS region from fecal samples collected before IFX and two and six weeks after the start of treatment. The response to IFX induction was determined by fecal calprotectin value < 100 µg/g at week six. The bacterial microbiota differed significantly between response groups, with higher relative abundance of butyrate-producing bacteria in responders compared to non-responders at baseline, validated by high predictive power (area under curve = 0.892) for baseline *Ruminococcus* and calprotectin. Additionally, non-responders had higher abundance of *Candida*, while responders had higher abundance of *Saccharomyces* at the end of the study. The gut microbiota composition in PIBD patients could predict response to IFX treatment in the future.

## Introduction

Inflammatory bowel diseases (IBD) including Crohn’s disease (CD), Ulcerative Colitis (UC) and IBD unclassified (IBDU) are chronic inflammatory diseases characterized by inflammation in the gastrointestinal tract. The incidence of pediatric IBD (PIBD) is increasing worldwide both in developing and developed countries^1^. At diagnosis PIBD often presents with similar symptoms to adults’, which may include diarrhea, abdominal pain, bloody stools and fever^2^. In addition, PIBD (especially CD) may cause malnourishment, leading to growth impairment, delayed puberty or even psychosocial problems^2^. Effective treatment is crucial in order to avoid these long lasting sequalae in patients with PIBD.

The current hypothesis for the pathogenesis of IBD is that the combination of genetic, environmental and microbial factors leads to altered gut permeability and abnormal and prolonged immune responses with the alteration of gut microbiota composition with predominance of pathobionts that contribute to gut inflammation^3^. The role of the fungal gut microbiota, the mycobiota, is not as established, but may also be characterized as dysbiotic^4^, with *Candida* spp. being possible drivers of the imbalance^5–7^. Additionally, it has been observed that *Saccharomyces* species are less abundant in patients with IBD compared to healthy controls (HC)^6^. Studies concerning the gut mycobiota in PIBD are few, and although the results vary somewhat between the studies, both *Candida* and *Malassezia* have been reported as increased in PIBD, as well as the phylum Basidiomycota, as reviewed recently^8^. Many studies have reported a reduction in the diversity of commensal bacteria, especially in the two most abundant phyla Bacteroidetes and Firmicutes, in adult patients with IBD^3^. Additionally, a reduced abundance of potentially anti-inflammatory genera such as *Roseburia*, *Bifidobacterium* and *Faecalibacterium* and an increased abundance of Proteobacteria, *Veillonellaceae*, *Pasteurellaceae, Fusobacterium*, and *Ruminococcus gnavus,* some of which have proinflammatory effects, have been reported in adult patients with IBD when compared to HC^3^. Accordingly, the bacterial microbiota of patients with PIBD has been found to differ from HC with an increased relative abundance of *Prevotella*^9^. A recent review regarding gut microbiota profiles in PIBD, encompassing 41 studies, reported an increase in *Enterococcus* and significant decrease in *Anaerostipes, Blautia, Coprococcus, Faecalibacterium, Roseburia, Ruminococcus, and Lachnospira* in PIBD compared to HC*,* and a decrease in alpha-diversity within majority of the included articles^10^.

There are multiple therapy choices for PIBD, including conventional medications such as 5-ASA, immunomodulators and steroids, and explored for novel treatment options, such as fecal microbiota transplantation^11^. Recent ECCO-ESPGHAN guideline recommends anti-tumor necrosis factor alpha (TNF-α), such as infliximab (IFX), in pediatric CD with a high risk of poor outcome, if serious growth delay is present, or EEN and corticosteroid treatment do not induce remission^12^. A recent ESPGHAN guideline for pediatric UC recommends IFX in chronically active of steroid dependent UC, uncontrolled by 5-ASA and thiopurines^13^. However, up to 50% of patients do not respond to IFX, and loss of response is frequent^14^. To this day, there are no clinical methods available to predict the response although many have studied the bacterial microbiota with the aim to find biomarkers for prediction of anti-TNF-α treatment response to improve the cost-effectiveness of the therapy^15–20^.

There are very limited studies on the mycobiota in patients with PIBD receiving IFX therapy, and the study approach differ from this study^21^. Previous studies regarding the treatment response and disease severity have reported a reduced relative abundance of *Faecalibacterium prausnitzii (F. prausnitzii)*^17,22^ and we recently found an increased relative abundance of *Candida* to correlate with poor treatment response to anti-TNF-α in adult patients with IBD^23^. It has been discovered that adult patients with IBD have an imbalanced Ascomycota and Basidiomycota ratio^24^ and a higher abundance of *Candida albicans* compared to HC^4^. In children, a low baseline abundance of *F. prausnitzii* was associated with a lack of mucosal healing and need of surgical treatment^9^. In our previous study regarding the fecal microbiota and the treatment response to anti-TNF-α in PIBD patients we discovered several bacteria as potential biomarkers predicting treatment response such as higher relative abundances of *Bifidobacterium*, *Clostridium colinum*, *Eubacterium rectale*, uncultured C*lostridiales*, and *Vibrio,* and a lower abundance of *Streptococcus mitis*^20^.

The aim of this study was to describe the gut microbiota in patients with PIBD introduced to anti-TNF-α medication IFX and to study whether the fecal fungal and bacterial microbiota profiles predict IFX treatment response. Also, we aimed to study the association between the gut fungal and bacterial microbiota. Improved understanding of the complex interactions between the fungi and bacteria of the intestine could provide new insight to dysbiosis seen in IBD, possibly even having impact on the adjustment of therapy.

## Results

Finding methods for predicting the response to IFX therapy to avoid unnecessary side effects and high cost, particularly for pediatric patients, is crucial. Here we have investigated the fungal and bacterial gut microbiota profiles with the aim to find predictive markers for IFX response.

### Patient characteristics and response to IFX

**Figure 1:**
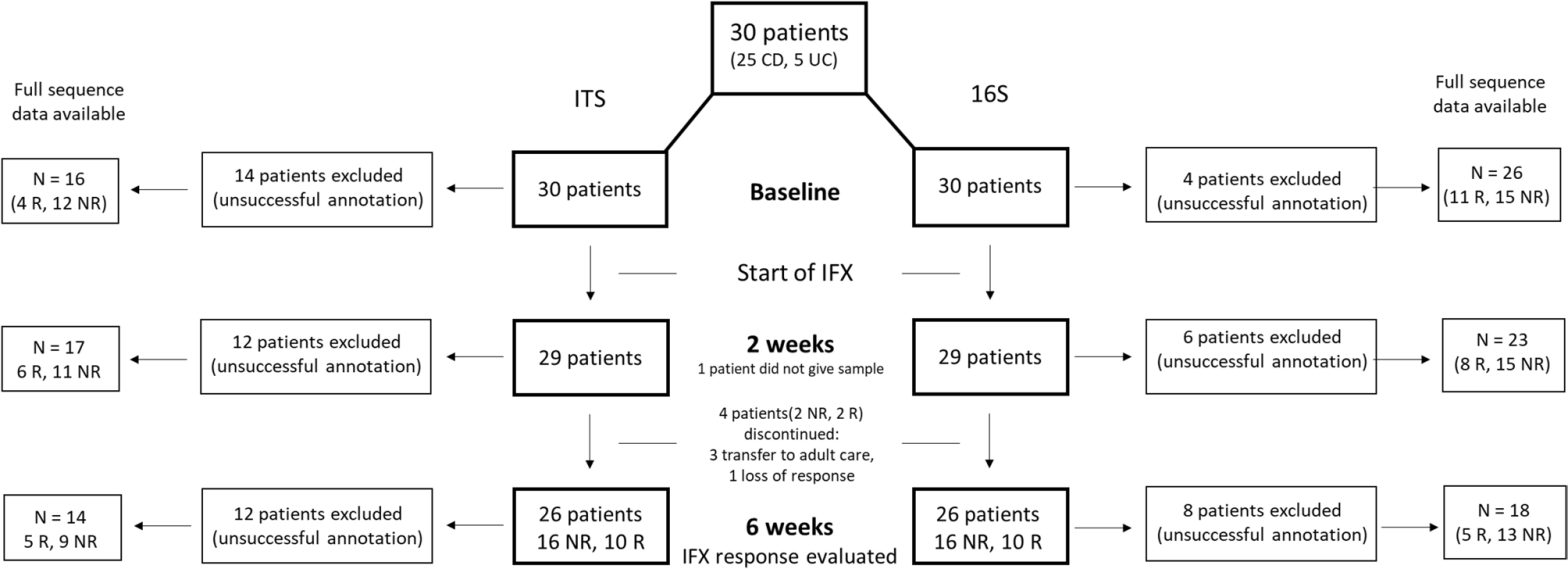
Overview of the study outline presenting the number of patients and samples at the three different timepoints (before start of treatment and 2- and 6 weeks post-treatment), response to infliximab (IFX) and reason of exclusion. R = responder, and NR = non-responder to IFX therapy.

In this study, a total of 30 patients with PIBD introduced to TNF-α antagonist IFX were included with a total of 85 samples collected before the start of IFX treatment and at two and six weeks of induction therapy. Out of the 30 patients, 27 patients with 68 samples were available for 16S sequencing, while 85 samples of 30 patients were available for ITS sequencing. 25 were diagnosed with CD and 5 with UC/IBDU. The background characteristics of the patients are shown in Table 1. At six weeks three patients were transferred to adult care. Therefore, the IFX response for three patients was determined by the fecal calprotectin value at week two since these patients terminated the study before week six (lack of third sample). One patient discontinued at week 2 due to loss of response and was considered a non-responder. In total 18 patients (60%) were NRs and 12 Rs. The number of patients and samples at each timepoint and the response to IFX of these for both the ITS and 16S data is presented in Fig. 1. IFX trough levels were available from 15 patients (9 NRs and 6 Rs) at 6 weeks post treatment. The median value was 4.6 mg/L (min–max = 0–11) for NRs and 11.05 mg/L (min–max = 0.79–10.1) for Rs. The trough levels did not differ significantly between the response groups.

**Table 1:** Patient characteristics.

Characteristics	n (%) or Median (min–max)	
No. of patients	30	
	12 R	18 NR
Male	8 (67)	13 (72)
CD	11 (92)	14 (78)
UC	1 (8)	1 (6)
IBD unclassified	0 (0)	3 (17)
Age at diagnosis, years	13 (6–16)	13 (6–16)
Age at IFX initiation, years	15 (9–18)	14 (6–17)
Anti-TNF-α naïve	9 (75)	17 (94)
Previous exposure to IFX*	3	1
Disease duration at recruitment, years	1.4 (0–4)	0.3 (0–6)
IBD surgery	0 (0)	2 (11)
Baseline fecal calprotectin value (µg/g)**	360 (7–1317)	769 (55–6293)
2-week fecal calprotectin value (µg/g)	25 (< 5–496)	456 (36–1876)
6-week fecal calprotectin value (µg/g)	41 (5–89)	399 (162–2142)
Baseline symptom index score^38^	1 (1–4)	1 (1–8)
Baseline VAS (disease impact on QOL)^38^	2 (1–4)	2 (1–6)
Baseline physicians’ global assessment^39^	2 (1–3)	2 (1–3)
**Concomitant medication at IFX initiation**		
Steroid	6 (50)	12 (67)
5-aminosalicylic acid	5 (42)	7 (58)
Azathioprine	7 (58)	3 (17)
Methotrexate	1 (8)	0 (0)
Ursodeoxycholic acid	1 (8)	1 (6)
Antibiotics***	5 (42)	9 (50)
*Saccharomyces boulardii* supplement****	0 (0)	2 (11)
Lactic acid bacteria supplement (regular use)	9 (75)	9 (50)

*CD* Crohn's disease, *UC* ulcerative colitis, *IBD* inflammatory bowel disease, *IFX* infliximab, *TNF* tumor necrosis factor.

*Time since exposure at baseline 10–26 months.

**Three patients had baseline fecal calprotectin value < 100 µg/g.

***Metronidazole, cephalosporin or amoxicillin in 12, other = 1.

****Two weeks prior to the study.

We had three patients with fecal calprotectin value < 100 at the start of the study (Table 1). Of the three patients with calprotectin < 100 at baseline the indication to start IFX treatment were steroid-dependency (n = 2) and perianal disease (n = 1).

### Overview of fungal and bacterial gut microbiota in patients with PIBD

#### Fungal gut microbiota

**Figure 2:**
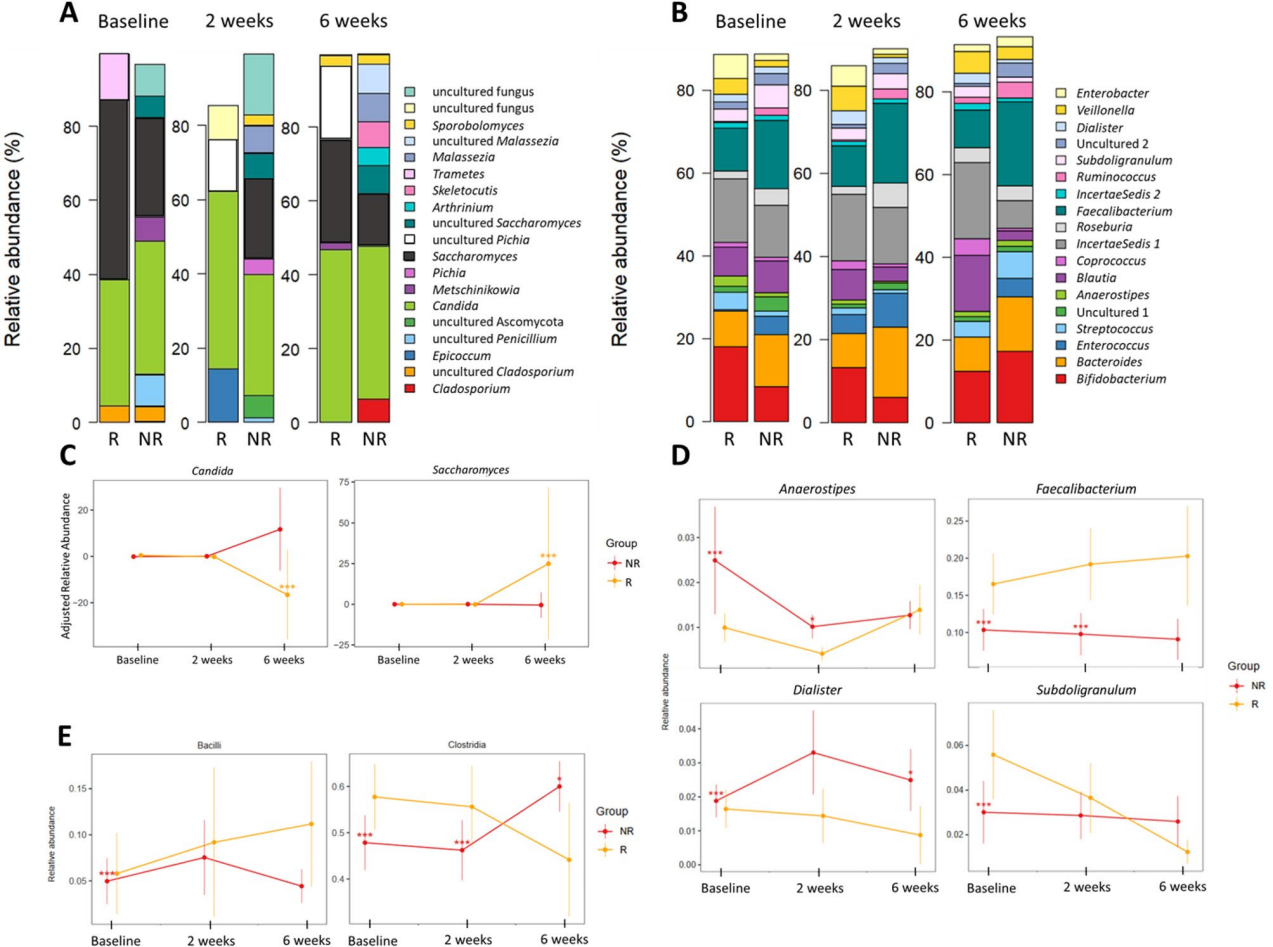
Overview of the fecal (**A**) fungal mycobiota and (**B**) bacterial microbiota composition before start of infliximab (IFX) therapy (baseline), two weeks and six weeks after treatment stratified by response to induction therapy to IFX. The plots present the most abundant genera, which are color-coded and shown on the right-side panel. (**C**) The standardized confounder-adjusted relative abundance of the fungal genera that significantly differed between response groups presented throughout the study and (**D**) the relative abundance of the bacterial genera and (**E**) classes that differed significantly at baseline between response groups presented throughout the study. R presents responders and NR presents non-responders. (*p FDR).

For fungal analyses, successful annotation with read count above 100 was achieved for 47 samples from 23 patients. Across all samples, the gut mycobiota was composed of Ascomycota (83%) and Basidiomycota (10%) and uncultured Eukaryota (7%). Altogether 33 genera were present in the gut mycobiota, and the most abundant genera were *Candida* (64%), followed by *Saccharomyces* (51%), uncultured fungus (20%) and *Cladosporium* (14%), presented in Fig. 2A. The prevalence for all genera is presented in Supplementary Table 1. At species level [*Candida albicans*] (38%) and [*Saccharomyces cerevisiae*] (22%) were most prevalent.

#### Bacterial gut microbiota

For bacterial analyses, a total of 68 samples from 27 patients were available. The fecal microbiota of the samples was composed of the phyla Firmicutes (68%), Actinobacteria (14%), Bacteroidetes (12%), and Proteobacteria (5%). The most abundant genera across all samples were *Faecalibacterium* (9.1%). *Bifidobacterium* (5.5%*), Blautia* (4.2%) and *Bacteroides* (4%)*.* The most prevalent genera at different timepoints are presented in Fig. 2B.

### Fungal and bacterial microbiota composition as markers for prediction of IFX response

#### Differences in fecal fungal microbiota according to IFX therapy response

The fecal mycobiota did not differ significantly between response groups at baseline. At two weeks after induction the order Saccharomycetales was more abundant in Rs (by 1.03-fold, *P* FDR < *0.001*). At six weeks the genus *Candida* [*C. albicans*] was more abundant in NRs (by 22-fold, *P* FDR < *0.001*) and *Saccharomyces* [*S. cerevisiae*] in Rs (by 1.9-fold, *P* FDR < *0.001*). This is presented in Fig. 2C where the standardized confounder-adjusted relative abundance of the genera *Candida* and *Saccharomyces* is plotted throughout the study. The fungal diversity and richness at the different timepoints stratified by response groups is presented in Supplementary Fig. 1A, although there were no significant differences between response groups.

#### Differences in response groups to IFX therapy in fecal bacterial microbiota

At baseline the bacterial microbiota composition differed between Rs and NRs. The classes Clostridia (1.2-fold, *P* FDR < *0.001*) and Bacilli (1.2-fold, *P* FDR < *0.001*) were more abundant in Rs, whereas the class Gammaproteobacteria (4.4-fold, *P* FDR < *0.001*) was more abundant in NRs. At genus level, the Rs had an increased relative abundance of *Faecalibacterium* (1.4-fold, *P* FDR < *0.001*) and *Subdoligranulum* (1.7-fold, *P* FDR < *0.001*), whereas the NRs had an increased abundance of *Dialister* (1.2-fold, *P* FDR < *0.001*) and *Anaerostipes* (2.3-fold, *P* FDR < *0.001*) when compared to the Rs. The statistically significantly differing genera are presented in Fig. 2D at all timepoints, and classes Bacilli and Clostridia are depicted in Fig. 2E. All statistically significantly differing bacterial taxa of other taxonomic levels are listed in Supplementary Table 2.

At six weeks after start of IFX treatment the Rs had an increased relative abundance of classes Actinobacteria (by 1.5-fold, *P* FDR = *0.041*) and Erysipelotrichia (by 3.3-fold, *P* FDR = *0.024*). At genus level, the Rs had decreased relative abundances of *Blautia* (by 5.6-fold, *P* FDR < *0.001*)*, Coprococcus* (by 6.3-fold, *P* FDR = *0.024*)*, Lachnospiraceae* (Insertae Sedis) (by 4.7-fold, *P* FDR = *0.0031*) and *Dialister* (by 11-fold, *P* FDR = *0.048*)*,* but an increased relative abundance of *Bifidobacterium* (by 2.0-fold, *P* FDR = *0.048*). All statistically significantly differing bacterial taxa of other taxonomic levels are listed in Supplementary Table 3.

**Figure 3:**
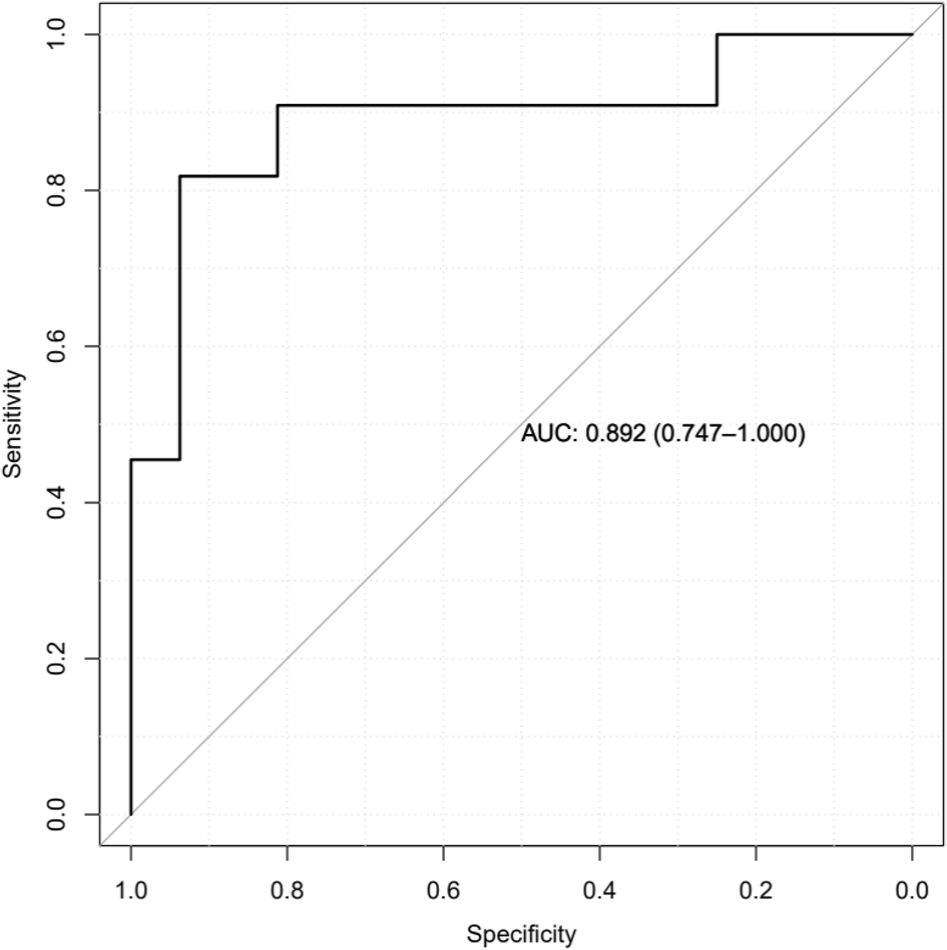
Receiver operating characteristic (ROC) curve to predict the response to infliximab therapy in patients with pediatric inflammatory bowel disease at baseline. The bacterial genus *Ruminococcus* and baseline fecal calprotectin values were included in the model. The area under curve (AUC) is indicated.

The bacterial diversity and richness at the different timepoints stratified by response groups is presented in Supplementary Fig. 1B. Neither bacterial diversity nor richness differed significantly between treatment response groups at any timepoints. The response to IFX therapy was predictable by the bacterial genus *Ruminococcus* combined with baseline fecal calprotectin values before start of treatment with AUC 0.892 respectively (Fig. 3). Additionally, the response was predicted by baseline *Ruminococcus* relative abundance alone (AUC 0.790).

### Disease duration, age, sex and antibiotics

#### Gut microbiota composition and disease duration before the start of IFX

Disease duration was defined as the time from diagnosis to the start of IFX treatment. In fecal mycobiota we observed no difference in the composition in relation to disease duration.

Regarding fecal bacterial microbiota, the patients with disease duration for more than a year (n = 14) had a lower relative abundance of the class Clostridia (by 1.2-fold, *P* FDR < *0.001*) at baseline. At genus level, this group of patients had a higher relative abundance of the genera *Faecalibacterium* (1.1-fold, *P* FDR < *0.001*) and *Subdoligranulum* (1.3-fold *P* FDR < *0.001*) when compared to patients with a disease duration less than a year (n = 14). All statistically significantly differing bacterial taxa of other taxonomic levels are listed in Supplementary Table 4.

#### Gut microbiota composition in age groups

The age of the patients varied from 6 to 18 years (median = 14) at the initiation of IFX therapy. To investigate the gut microbiota composition according to age stratification we divided the patients into older and younger than 12 years (an arbitrary cut-off for prepubertal and pubertal patients), with 8 patients being younger than 12 years at IFX therapy initiation. The analyses were done at baseline.

In the fungal microbiota we observed that the genus *Saccharomyces* [*S. cerevisiae*] was significantly more abundant (*P* FDR < *0.001*, by twofold) in patients younger than 12 years of age.

In the bacterial microbiota, we discovered that the patients older than 12 years had an increased relative abundance of the class Clostridia (by 1.1-fold, *P* FDR < *0.001*), but a decrease in Bacilli (by 2.8-fold, *P* FDR < *0.001*). At genus-level, the patients older than 12 years had a lower relative abundance of *Dialister* (by 1.9-fold, *P* FDR < *0.001*). All statistically significantly differing bacterial taxa of other taxonomic levels are listed in Supplementary Table 5.

#### Gut microbiota composition difference between sex

There was a significant difference in mycobiota composition between sex with a higher abundance of the class Saccharomycetes (by 2.9-fold, *P* FDR = *0.04*) and a lower abundance of the genus *Saccharomyces* [*S. cerevisiae*] (by 2.1-fold, *P* FDR < *0.001*) in females compared to males at baseline.

At baseline we observed a higher relative abundance of the class Clostridia (by 1.1-fold, *P* FDR < *0.001*) in males, when compared to females. At genus level, females had significantly higher relative abundances of *Faecalibacterium* (2.1-fold, *P* FDR < *0.001*), but a lower relative abundance of *Dialister* (by 1.5-fold, *P* FDR < *0.001*). All statistically significantly differing bacterial taxa of other taxonomic levels are listed in Supplementary Table 6.

#### The effect of antibiotics on the microbiota composition

We discovered that patients who had received antibiotics during the prior month to sampling had a higher relative abundance of the fungal genus *Saccharomyces* [*S. cerevisiae*] (by twofold, *P* FDR < *0.001*) at baseline. Additionally, the antibiotic-treated patients had a lower abundance of Clostridia (by 1.2-fold, *P* FDR < *0.001)*, but an increased abundance of Bacilli (by threefold, *P* FDR < *0.001*). At genus-level, patients receiving antibiotics had a significantly reduced abundance of *Faecalibacterium* (by 4.2-fold, *P* FDR < *0.001*) compared to those who did not receive antibiotics one month prior to sampling. All statistically significantly differing bacterial taxa of other taxonomic levels are listed in Supplementary Table 7.

### Correlation between fungal and bacterial microbiota composition

Correlations between fungal and bacterial microbiota were eligible to be calculated for 5 Rs and 13 NRs at baseline and the correlations differed between the response groups, values at baseline are presented in Supplementary Tables 8 and 9.

In Rs *Saccharomyces* correlated positively with *Akkermansia* (r = 0.89, *P* = *0.04*) and *Candida* correlated positively with *Lactococcus* (r = 0.92, *P* = *0.03*) at baseline.

Spearman correlations between fungal and bacterial genera in NRs are presented in Table 2.

**Table 2:** Statistical tests assessing Spearman correlations between relative abundance of fungal and bacterial genera were performed at baseline (introduction to infliximab) in pediatric patients with inflammatory bowel disease who turned out to be non-responders to infliximab at the end of induction therapy (week six).

Bacterial genus	Fungal genus	Spearman correlation (r)	*P* value
*Bacteroides*	*Botrytis*	0.63	0.02
*Barnesiella*	Uncultured *fungus*	0.57	0.04
*Blautia*	Uncultured *fungus*	0.68	0.01
*Collinsella*	*Candida*	0.60	0.03
*Parasutterella*	*Botrytis*	− 0.59	0.03
*Peptostreptococcus*	*Saccharomyces*	− 0.59	0.03
*Prevotella*	*Saccharomyces*	− 0.58	0.04
IncertaeSedis	Uncultured *Cladosporium*	− 0.71	0.006
IncertaeSedis	Uncultured *Penicillium*	0.56	0.047
*Lactobacillus*	Uncultured *Cladosporium*	0.706	0.007
*Enterococcus*	Uncultured *Penicillium*	0.56	0.047
*Phascolarctobacterium*	Uncultured *Penicillium*	0.60	0.03
*Klebsiella*	Uncultured *Saccharomyces*	0.63	0.02

## Discussion

In this study, we explored the fungal and bacterial gut microbiota in pediatric patients with IBD, who received anti-TNF-α medication IFX with the aim to find microbiota markers for prediction of treatment response. We demonstrate differences in the gut microbiota compositions between response groups to IFX therapy in PIBD, further validated by high predictive power for the bacterial microbiota. Additionally, we found differences in the interkingdom interactions between the response groups. Together these results suggest that intestinal microbes have potential as biomarkers for prediction of treatment response in patients with PIBD, that previously have been investigated from the bacterial microbiota profile but are largely unexplored for the fungal microbiota.

Although being less abundant than bacteria^25^, the fungal species seem to have a significant role in IBD, with a composition characterized by a higher abundance of Basidiomycota and lower abundance of Ascomycota compared to HC^24^. This is observed as an increase of *Candida* and a decrease of *Saccharomyces* and *Malassezia* in IBD^24^. The gut mycobiota of patients with PIBD consisted of the phyla Ascomycota and Basidiomycota across all samples, as previously observed^8,24^. The most abundant genus was *Candida*, followed by *Saccharomyces*, also agreeing with previous studies^24^. In pediatric studies, *Candida* has been observed to associate with PIBD and with disease intensity^5,24,26^.

In younger, in male and in antibiotic-treated patients, the relative abundance of *Saccharomyces* was higher. The number of female patients was low (less than 30% of all patients), and most were prepubertal. Using age-grouping below and above 12 years is somewhat arbitrary due to not considering factors such as females and males reaching puberty at different ages, and the impact of IBD on the age of reaching puberty. The species *S. boulardii* is characterized with anti-inflammatory properties and is used as a probiotic^27^, however only two of our patients used such probiotics two weeks prior to the study, of which both had *Saccharomyces* present in the mycobiota. Antibiotics cause a decrease in bacteria, which in turn causes less competition to fungal species^28^. If fungi play a role in IBD disease progression, the use of antibiotics may have unpredicted consequences as fungi will benefit from it.

The bacterial microbiota consisted of the phyla Firmicutes*,* Actinobacteria*,* Bacteroidetes and Proteobacteria*,* accounting up to 99% of all bacteria. Similar bacterial composition has been discovered in adult studies, both within HC and IBD patients, where these four phyla accounted from 95 to 98% of all reads^15^.

Longer disease duration associated with a reduced relative abundance of the class Clostridia, but an increase in Clostridia genera, namely *Faecalibacterium* and *Subdoligranulum. Faecalibacterium* and *Subdoligranulum* both produce butyrate which has an anti-inflammatory effect by upregulating the secretion of IL-10, an anti-inflammatory cytokine, and strengthening the intestinal barrier^26,29–31^. This suggests a decreased inflammation in patients for whom the disease duration was more than a year. Active inflammation in the gut has been noted to alter the intestinal microbiota and change during remission in response to anti-TNF-α^7,15,16,22^.

Finally, patients who received antibiotics two weeks prior to the study had a significantly reduced abundance of *Faecalibacterium,* an important anti-inflammatory bacterium, as stated above. Either the disease is more severe and perhaps fistulizing and therefore antibiotics have been used as a treatment, or the use of antibiotics has severely disturbed the intestinal homeostasis towards a more inflammatory state.

Few have investigated the mycobiota in PIBD^7,8,26^, and to our knowledge there is only one study investigating it for prediction of response to IFX treatment^21^, in which a metagenomic approach was used to investigate the microbiome, making it complicated to compare to this study. Here, the mycobiota was comparable between response groups at baseline, agreeing with the previous study^21^. However, *Candida* was significantly elevated in NRs while *Saccharomyces* was elevated in Rs at six weeks after therapy. This could indicate that the gut mycobiota of Rs shifts towards a healthier composition, since it has been observed that in HC *Saccharomyces* is increased^24^, while *Candida* is associated with disease progression in both adult and pediatric patients with IBD^4,23,26^. In adult IBD, we recently found that *Candida* was elevated in adult NRs to IFX prior to therapy initiation^23^. IBD is different in and pediatric patients, and in our adult study (median age 31 years) there were more UC patients compared to CD, different drugs were used in addition to IFX, and the male to female ratio was different; all of which might cause the differing results^23^.

The bacterial fecal microbiota in PIBD patients has been studied previously^7,9,15–17,20,21^. The methodologies used in these studies vary, which must be considered when comparing the results. We found that treatment response was associated with a microbiota characterized by bacteria capable of anti-inflammatory butyrate production, which might help reach and maintain remission. Rs had a higher relative abundance of the classes Clostridia and Bacilli, belonging to the phylum Firmicutes and the order Clostridiales at baseline*.* These results are supported by studies on both pediatric and adult cohorts investigating the response to biological therapy in the fecal microbiota^15,20,23^. In a previous study on adults, the relative abundance of Clostridiales increased during IFX treatment, resulting in a microbiota resembling HC, indicating that Clostridiales re-enrichment correlated with remission^15^. In PIBD, we previously discovered that the relative abundance of uncultured Clostridiales predicted treatment response to IFX^28^. Differences between Rs and NRs at such a high taxonomic level indicate a deeper dysbiosis in NRs (compared to Rs), since the relative abundance of Clostridiales is reduced in IBD^7,15,20^.

An increased baseline abundance of *Faecalibacterium* has been associated with response to biological therapy in adult and pediatric IBD^9,15,17,22^*.* Additionally, an increase in *Faecalibacterium* and another butyrate producing genus, *Roseburia*, has been associated with response to anti-TNF treatment^7,15,16,22^. The higher relative abundance of Clostridiales, *Faecalibacterium* and *Subdoligranulum,* in Rs reflects gained similarity to microbiota in HC along therapeutic response^7,9,16,20^. In Rs, we also discovered an increase in the class Bacilli and order Lactobacillales and a reduced relative abundances of the genus *Dialister.* In our previous study on adults, we observed *Dialister* to be decreased in UC in Rs to IFX before the start of treatment^23^.

In most studies the microbiota of Rs to anti-TNF-α has been associated with a baseline microbiota closer to HC and shifting during treatment even more towards that of HC^7,9,15–17^. Unexpectedly, we discovered that the Rs had a decreased relative abundance of *Dialister, Blautia* and *Coprococcus* at six weeks after the start of IFX treatment and all of these have been reported decreased in PIBD^7,16,20^, especially in those patients with a more profound dysbiosis^7^. However, we observed *Bifidobacterium* to be increased in the Rs at six weeks potentially balancing the dysbiosis caused by the lack of the likely beneficial *Dialister, Blautia* and *Coprococcus.*

A higher baseline diversity and an increase in microbial diversity during treatment has been associated with response to anti-TNF-α treatment in PIBD^7,20^. However, we did not discover statistically significant differences in fungal or bacterial diversities between treatment response groups at any timepoints.

The predictive power of the microbiota composition was further studied by the performance of the models created from the genera that we determined to differ significantly between response groups by the PathModel function. The AUC indicated high predictive power for the bacterial model. *Ruminococcus,* a bacterial genus that we used in the model, has previously been associated with response to anti-TNF-α medication^22^ with a decrease in Rs, in line with our results. In our previous study done on adult patients with IBD we found that the family unknown *Ruminococcaceae* was predicting the response at baseline with high AUC^23^. In the model for ROC curve analyses baseline fecal calprotectin value together with *Ruminococcus* was also predicting response. Fecal calprotectin is a neutrophil-derived biomarker of intestinal inflammation that is used in the evaluation of therapeutic responses particularly in pediatric patients where invasive methods such as biopsy are not preferable^32^. *Candida* predicted response in our previous study done on adult IBD patients^23^. Although a significant difference was not observed between response groups at baseline here, *Candida* was significantly more abundant in NRs at the end of the study.

The interaction between fungi and bacteria in the gut may have a significant role in the pathology of IBD^23,24^. Interestingly, we found that the interkingdom correlations differed between response groups. This difference was characterized by bacteria that previously have been shown to have beneficial roles in patients with IBD that correlated with *Candida* and *Saccharomyces* in Rs, while bacteria that have been associated with dysbiosis in IBD correlated with *Candida* and *Saccharomyces* in NRs. In Rs we found positive correlations between the abundance of *Candida* and *Lactococcus,* and between *Saccharomyces* and *Akkermansia*. *Lactococcus* has previously been associated with response to anti-TNF-α^7^ and lower abundances of *Akkermansia* has been associated with more severe inflammation in adult patients with UC^33^. In NRs we found *Candida* to correlate positively with *Collinsella*. As previously stated, *Candida* has been associated with non-response to anti-TNF-α^23^, while *Collinsella* has been associated with penetrating disease in PIBD^34^. Further, the abundance of *Saccharomyces* correlated negatively with *Peptostreptococcus* and *Prevotella* while unknown *Saccharomyces* correlated positively with *Klebsiella* in NRs. In adult patients with IBD, *Peptostreptococcus* was increased in NRs to anti-TNF-α^19^. High *Prevotella* and *Klebsiella* relative abundance is associated with dysbiosis^35^. Improved understanding of the interplay and relation of myco- and microbiota is needed to find out whether it is possible to modify the intestinal balance of species towards a more anti-inflammatory form to improve therapeutic responses.

The strengths of this study are that the samples were immediately frozen after sampling, ensuring good quality and exceptionally low loss of follow up. As a limitation, we had a relatively limited number of patients, as in most pediatric studies. However, this cohort is, to our knowledge, the largest one studying both the fungal and bacterial gut microbiota as predictive markers for IFX response in PIBD patients thus far. The problem with zero-inflation considering the ITS data is one not only present in this study, but in most studies concerning ITS data. Here it is evident in the number of samples with no detectable fungi or unsuccessful annotation. This could be either biological or methodological. Finally, the predictive taxa of fungi and bacteria found need to be validated in an independent, larger cohort to be applicable in clinical settings.

To conclude, we found significant differences between response groups to IFX medication in the fungal and bacterial microbiota composition of patients with PIBD, seen in the bacterial microbiota already before the start of IFX therapy. Non-responders had lower abundance of butyrate-producers such as bacteria in the Clostridia class, particularly the genera *Faecalibacterium* and *Subdoligranulum* before start of IFX therapy. The response was predictable at baseline by *Ruminococcus* (belonging to class Clostridia) and baseline fecal calprotectin values. Additionally, *Candida* was more abundant in NRs, while *Saccharomyces* was more abundant in Rs after IFX therapy. Together with previously published data, our results indicate that gut microbiota composition is a valuable resource in prediction of anti-TNF-α treatment response.

## Methods

### Study design

Patients with IBD introduced to IFX therapy at the Children´s Hospital, University of Helsinki were recruited to the study between the years 2011 and 2016. The participants were requested to collect a stool sample for study purposes before the start of IFX infusion and at two- and six-weeks infusions concurrent to obtaining the routine sample for fecal calprotectin. Samples were immediately frozen at home in –20 °C and transported to laboratory where they were stored at − 80 °C until processing.

### IFX response

The response to IFX was evaluated by fecal calprotectin value at 6 weeks after IFX therapy initiation. IFX response (R) was determined by fecal calprotectin value below 100 µg/g and non-response (NR) with a value above 100 µg/g^36^. The cut-off value of < 100 µg/g has a specificity of 0.92 and negative prediction value of 95%^36^ and predicts transmural healing with high accuracy^37^. We assessed clinical disease activity using a validated tool with symptom score and visual analogy scale (VAS) from 1 to 7, measuring the impact of disease to quality of life (QOL)^38^. Symptom score contained 5 questions (total score 0–15 points, higher score being worse) regarding general well-being, abdominal pain, nocturnal and daily bowel movements, and the presence of blood in the stools. This index is applicable to adult and pediatric patients with IBD and in UC as well as in Crhon’s disease^38^. We also did physicians global assessment (PGA) of the general activity of the disease ranging from 1 to 3, with 3 being the most active disease^39^. All measurements of disease activity are listed in Table 1.

### DNA extraction from fecal samples and preparation of sequencing libraries

The samples were thawed, and DNA was extracted by using a repeated bead beating method as previously described^40^. The DNA from fungal and bacterial organisms was amplified in PCR reactions targeting the 16S rRNA gene and ITS1 regions in separate reactions as previously^23^. The fecal bacterial composition was determined by sequencing the V3-V4 region of the 16S rRNA gene with primers 341FWD 5′-CCTACGGGNGGCWGCAG-3′ and 785REV 5′-GACTACHVGGGTATCTAATCC-3′^41^. The fecal fungal composition was analyzed by sequencing the ITS1 region using primer pair ITS1F (FWD, CTTGGTCATTTAGAGGAAGTAA) and ITS2 (REV, GCTGCGTTCTTCATCGATGC)^38,42^. The libraries for sequencing were prepared as previously described^23^. Illumina MiSeq paired-end sequencing was performed in Functional Genomics Unit, University of Helsinki, Helsinki, Finland in separate runs. The ITS and 16S rDNA sequences are available at the ENA database (ITS accession number: PRJEB50351, 16S accession number: PRJEB50380).

### Analysis of sequencing data

The 16S MiSeq sequencing data was analyzed using the R package mare^43^. The median number of reads obtained per sample was 38 037 (range 21 743–94 758) for bacteria. Default parameters were used in processing of only forward reads truncated to 150 bases by using the function ProcessReads in mare. Reads below the abundance of 0.002% were discarded. After the processing the median read count was 29 254 (range 16 051-73 478). All samples from the 27 patients included were annotated successfully and included in further analyses. Annotation to bacterial taxa was done using USEARCH^44^ and the reads were annotated to the database SILVA v3v4^45^, restricted to gut-specific bacteria.

The ITS data was processed according to the ITS pipeline included in DADA2^46^. Annotation was done as described previously^23^ by annotating the amplicon sequence variants (ASVs) with BLAST^47^. The median number of reads per sample was 58,700 before pre-processing. After annotation the average read count was 4000 reads. Successful annotation to fungal taxa was obtained from 59 samples (69%) from 27 patients (out of 30 patients available for ITS sequencing). Annotations at species-level were the best hits based on the annotations to BLAST^47^ and are included in brackets for fungi.

### Statistical analysis

Both for the 16S rRNA gene and ITS relative abundance data, the package mare^43^ was used for analysis with the program R using the packages vegan^48^, MASS^49^, nmle^50^, and pROC^51,52^. P-values for taxon-specific differences were corrected for false discovery rate (FDR; Benjamini–Hochberg^53^). The data-based selection of background variables was based on PERMANOVA and all background variables presented in Table 1 were evaluated. Those that were statistically significantly associated with the fungal and bacterial microbiota composition were used as co-variates to adjust the analyses at the three different timepoints. Generalized linear models with negative binomial distribution (glm.nb) from the MASS package^49^ and Generalized Least Squares (gls) from the nlme package^50^ were used to analyze differences in response groups and IBD subtypes for both the fungal and bacterial microbiota data. In all bacterial analyses at the three timepoints, IBD subtype, sex, previous antibiotics (less than one month prior to sample), the use of corticosteroid-medication and age (grouped to below and over 12 years) were used as confounders. In bacterial analyses 0.1 was used as minimum prevalence and 0.01 as minimum abundance. In all fungal analyses at the three different timepoints, age, sex, IBD subtype and antibiotics were used as confounders. Due to zero-inflation in the fungal data, only taxa with a prevalence above 50% were included in the analysis. In all analyses the IBDU patients were included with the UC patients. The diversity was calculated as the inverse Simpson diversity index and richness as the number of operational taxonomic units.

PathModel function in R package mare^43^ was used to identify the genera that together predicted response and to find the ideal glm model^49^ to fit the data. The performance of the model was tested and visualized by performing receiver operating characteristic (ROC) analysis with the pROC package^51,52^.

The association between fecal fungal and bacterial genera was done by calculating Spearman correlations and p values, with phyloseq objects created for both ITS and 16S rRNA gene amplicon data^54^. To obtain a list of uniquely annotated ASVs, the tax_glom function from phyloseq^54^ was used for agglomeration over the "genus" taxonomic rank. The resulting list was filtered to select taxa with over 100 reads across the samples. No co-variates were used to adjust the correlations.

### Ethics statement

The study was approved by the ethical committee of the Hospital District of Helsinki and Uusimaa (extension approved in 2014 to study, approved with a diary number 183/13/03/03/2011). All participants (or their guardians) signed an informed consent. All methods were performed in accordance with relevant guidelines and regulations.

## Supplementary Information

Supplementary Information 1.

Supplementary Information 2.
