# Supplementary material for: The gut fungal and bacterial microbiota in pediatric patients with inflammatory bowel disease introduced to treatment with anti-tumor necrosis factor-α
Source: Sci Rep. 2022 Apr 22;12:6654. doi: 10.1038/s41598-022-10548-7 (PMC9033777; doi:10.1038/s41598-022-10548-7)
Supplement: Supplementary file 1 — Supplementary Information 1. [file 41598_2022_10548_MOESM1_ESM.pdf]

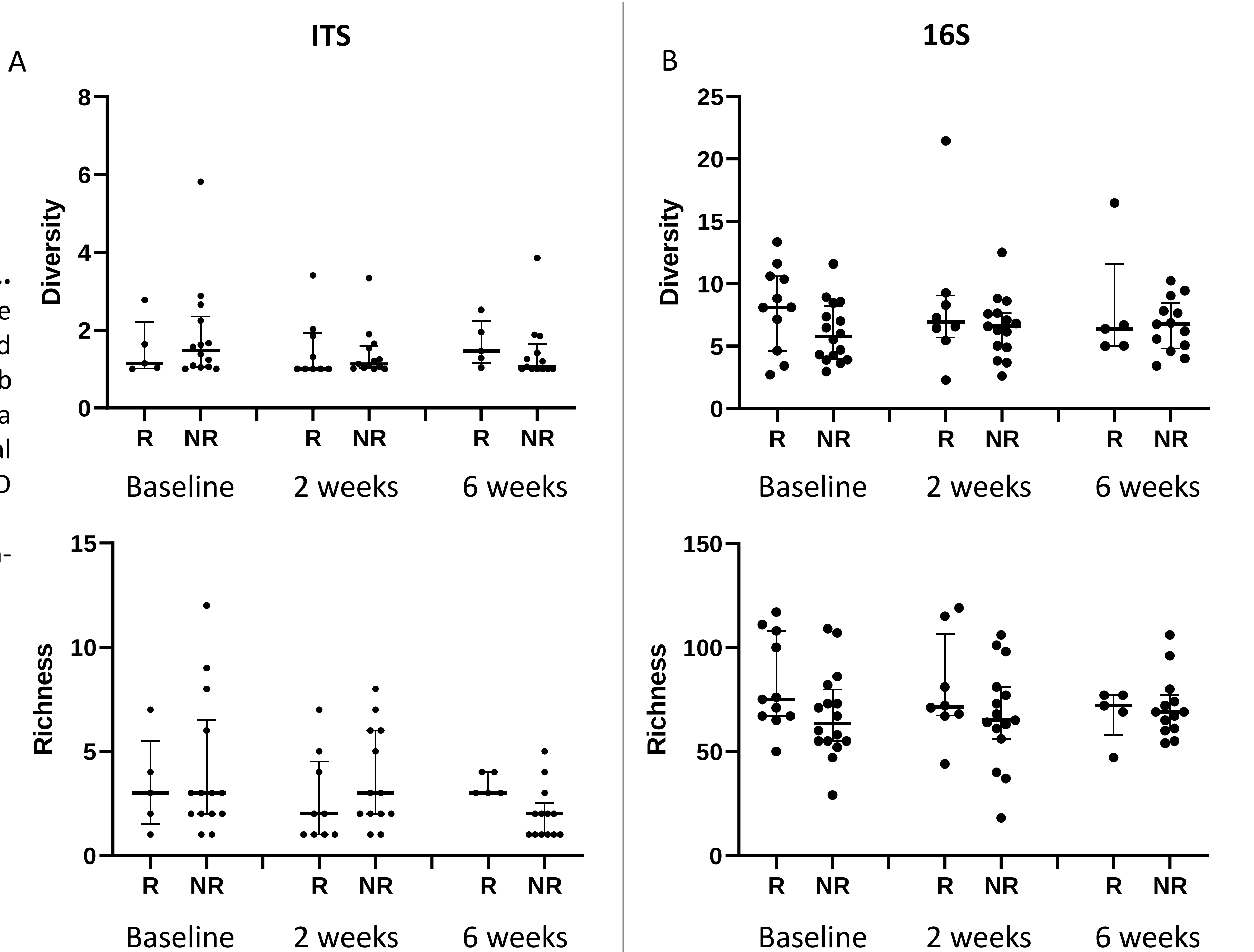

Supplementary Figure 1. Diversity and richness at the different timepoints stratified by response to infliximab therapy of A. gut mycobiota and B. gut bacterial microbiota in pediatric IBD patients.
R = responder and NR = non-responder to infliximab
